# Metagenomics reveals global-scale contrasts in nitrogen cycling and cyanobacterial light-harvesting mechanisms in glacier cryoconite

**DOI:** 10.1186/s40168-022-01238-7

**Published:** 2022-03-23

**Authors:** Takumi Murakami, Nozomu Takeuchi, Hiroshi Mori, Yuu Hirose, Arwyn Edwards, Tristram Irvine-Fynn, Zhongqin Li, Satoshi Ishii, Takahiro Segawa

**Affiliations:** 1 Department of Informatics, National Institute of Genetics, Shizuoka, Japan; 2 Advanced Genomics Center, National Institute of Genetics, Shizuoka, Japan; 3 Department of Earth Sciences, Graduate School of Science, Chiba University, Chiba, Japan; 4 Department of Applied Chemistry and Life Science, Toyohashi University of Technology, Aichi, Japan; 5 Institute of Biological, Environmental & Rural Sciences (IBERS), Aberystwyth University, Aberystwyth, UK; 6 Interdisciplinary Centre for Environmental Microbiology, Aberystwyth University, Aberystwyth, UK; 7 Department of Geography and Earth Sciences, Aberystwyth University, Aberystwyth, UK; 8 State Key Laboratory of Cryospheric Sciences/Tien Shan Glaciological Station, Northwest Institute of Eco-Environment and Resources, Chinese Academy of Sciences, Lanzhou, China; 9 Department of Soil, Water and Climate, University of Minnesota, St. Paul, MN USA; 10 BioTechnology Institute, University of Minnesota, St. Paul, MN USA; 11 Center for Life Science Research, University of Yamanashi, Yamanashi, Japan

**Keywords:** Glacier ecosystem, Cryoconite, Metagenomics, Denitrification, *Cyanobacteria*, Phycobilisome, Chromatic acclimation

## Abstract

**Background:**

Cryoconite granules are mineral–microbial aggregates found on glacier surfaces worldwide and are hotspots of biogeochemical reactions in glacier ecosystems. However, despite their importance within glacier ecosystems, the geographical diversity of taxonomic assemblages and metabolic potential of cryoconite communities around the globe remain unclear. In particular, the genomic content of cryoconite communities on Asia’s high mountain glaciers, which represent a substantial portion of Earth’s ice masses, has rarely been reported. Therefore, in this study, to elucidate the taxonomic and ecological diversities of cryoconite bacterial consortia on a global scale, we conducted shotgun metagenomic sequencing of cryoconite acquired from a range of geographical areas comprising Polar (Arctic and Antarctic) and Asian alpine regions.

**Results:**

Our metagenomic data indicate that compositions of both bacterial taxa and functional genes are particularly distinctive for Asian cryoconite. Read abundance of the genes responsible for denitrification was significantly more abundant in Asian cryoconite than the Polar cryoconite, implying that denitrification is more enhanced in Asian glaciers. The taxonomic composition of *Cyanobacteria*, the key primary producers in cryoconite communities, also differs between the Polar and Asian samples. Analyses on the metagenome-assembled genomes and fluorescence emission spectra reveal that Asian cryoconite is dominated by multiple cyanobacterial lineages possessing phycoerythrin, a green light-harvesting component for photosynthesis. In contrast, Polar cryoconite is dominated by a single cyanobacterial species *Phormidesmis priestleyi* that does not possess phycoerythrin. These findings suggest that the assemblage of cryoconite bacterial communities respond to regional- or glacier-specific physicochemical conditions, such as the availability of nutrients (e.g., nitrate and dissolved organic carbon) and light (i.e., incident shortwave radiation).

**Conclusions:**

Our genome-resolved metagenomics provides the first characterization of the taxonomic and metabolic diversities of cryoconite from contrasting geographical areas, highlighted by the distinct light-harvesting approaches of *Cyanobacteria* and nitrogen utilization between Polar and Asian cryoconite, and implies the existence of environmental controls on the assemblage of cryoconite communities. These findings deepen our understanding of the biodiversity and biogeochemical cycles of glacier ecosystems, which are susceptible to ongoing climate change and glacier decline, on a global scale.

**Video abstract**

**Supplementary Information:**

The online version contains supplementary material available at 10.1186/s40168-022-01238-7.

## Background

Glaciers and ice sheets are a biome rich in a variety of cold-adapted microbes [1, 2]. At the surface of glaciers, a hotspot for biogeochemical cycling is cryoconite, which is a dark-colored, granular aggregate composed of mineral particles and microbes dominated by metabolically active filamentous *Cyanobacteria*, along with algae, heterotrophic bacteria, and meiofauna [3–5]. Within cryoconite, various biogeochemical reactions are known to occur including carbon fixation, assimilation/dissimilation of inorganic nitrogen species, and biosynthesis of organic compounds [6, 7]. In addition, due to its light-absorbing dark color, the growth and distribution of cryoconite at the glacier surface can enhance ice melt [8, 9].

Glaciers are distributed over a broad range of geographically and climatologically contrasting regions: from high-latitude Polar deserts to maritime or continental alpine regions at lower latitudes. Cryoconite has been observed in all of these glacier regions [4]. However, previous studies based on 16S rRNA gene sequencing have demonstrated that the community structure of cryoconite differs across regions [10]. A phylogenetic study of 16S rRNA genes of cryoconite-constituting *Cyanobacteria* also indicated their distinct distribution between Polar and Asian alpine glaciers [11]. Such geographical differences in the bacterial assemblage of cryoconite suggest that its metabolic potential may also be geographically variable. Because cryoconite microbiota contribute to the nutrient cycling at the local glacier surface and in wider downstream ecosystems [12], it is important to understand the geographical diversity of the metabolic potential of cryoconite communities as the glaciers upon which they reside face an uncertain future as Earth’s climate is forecast to change [13, 14].

Glaciers in High-Mountain Asia (HMA) represent the largest portion of the Earth’s ice masses after the Polar ice sheets, but differ from their Polar counterparts in many ways, including their altitude, high input of dust from inland deserts, and anthropogenic pollutants from urban areas [15, 16]. Genetic and physiological information on cryoconite communities from Polar regions has been widely reported [17–19]. However, ecological and functional insight into microbial life within HMA cryoconite remains limited.

To illuminate the geographical diversity of cryoconite bacterial microbiomes, we collected cryoconite samples from a variety of glaciers around the globe, targeting sites in Polar and HMA regions, and then conducted shotgun metagenomic sequencing. In cryoconite, *Cyanobacteria* are keystone taxa because they are the dominant primary producers and their filamentous nature drives the granulation of cryoconite [20–22]. Therefore, we explored the genomic features of *Cyanobacteria* in our analysis.

To our knowledge, this is the first report on the global-scale comparative metagenomics of cryoconite. Our study revealed the distinct bacterial communities in cryoconite between Polar and high mountain Asian glaciers, thereby providing insight into the environmental controls on the bacterial assemblage of cryoconite, and expanding our knowledge on the global diversity of glacier ecosystems, which play an important role in global biogeochemical cycles but are susceptible to ongoing climate change and glacier ice loss.

## Materials and methods

### Sample collection

Cryoconite samples were collected from 21 sites distributed over 11 glaciers spanning from Polar to HMA regions: Warszawa Icefield (designated as JB) in King George Island, Antarctic Peninsula; Tyndall Glacier (Tyn) in Chilean Patagonia; Qaanaaq Ice Cap (Qaa) in Greenland Ice Sheet; Austre Brøeggerbreen (SbAb3) and Foxfonna Glacier (SbFxS1) in Svalbard; Gulkana Glacier (Gul) in Alaska; Yala Glacier (Yl) in Himalaya; Fedchenko Glacier (Fed) in Pamir; Grigoriev Ice Cap (Kir) and Urumqi No. 1 Glacier (Umq) in Tien Shan; and Qiyi Glacier (QiS4) in Qilian Shan regions (Additional file 1: Table S1). At each of the sites, we collected cryoconite from five randomly selected, discrete locations, which were positioned away from the glacier ice margins. Samples were subsequently kept frozen during transport and stored at − 80 °C until the DNA extraction and spectral analysis was undertaken.

### DNA extraction and sequencing

DNA was extracted from cryoconite samples (0.2–0.4 g in wet weight) as described previously [23] except that the bead-beating was done using a Multi-beads shocker at 2500 rpm for 30 s (Yasui Kikai, Osaka, Japan) in 2-ml Matrix-E tubes (MP Biomedicals, Santa Ana, CA, USA). All DNA extractions were performed in a Class 100 clean bench (MHE-130AB3; PHCbi, Japan). Sample replicates (*n* = 5 locations within each site) were combined to obtain a sufficient amount of DNA for subsequent analyses. An aliquot of DNA template (600–1000 ng) was sheared to a peak target size of 350 bp and 550 bp for the Illumina HiSeq and MiSeq sequencing, respectively, by using a Covaris S220 Focused-Ultrasonicator system (Covaris, Woburn, MA, USA). Sequencing libraries were constructed with a TruSeq DNA PCR-Free Library Preparation kit (Illumina, San Diego, CA, USA) or KAPA HyperPrep kit PCR-free (Roche, Basel, Switzerland). Constructed libraries were then size-selected by agarose gel electrophoresis and purified with the NucleoSpin Gel and PCR Clean-up kit (Takara Bio, Shiga, Japan). Sequencing reactions were carried out on a MiSeq platform (2 × 300 or 2 × 250 cycles) at the National Institute of Polar Research, Japan; a HiSeq 2500 platform (2 × 100 cycles) at the National Institute of Genetics, Japan; and a HiSeq 2000 and HiSeq X Ten platform (2 × 100 cycles and 2 × 150 cycles, respectively) at the Beijing Genomics Institute, China.

### Sequence data processing, assembly, and annotation

Adapters and terminal of low-quality sequences were first removed by using fastp v0.19.5 [24] with the following parameter settings: n_base_limit = 0, cut_by_quality5/3 = TRUE, low_complexity_filter = TRUE, and length_required = 30 and 100 for HiSeq and MiSeq reads, respectively. Reads with low complexity were further removed by using prinseq-lite [25] with the following parameters: lc_method = dust and lc_threshold = 15. The resulting high-quality read pairs originating from the same sample were assembled into contigs by using MEGAHIT v1.1.3 [26]. Sequence reads from Qaa_1 and Qaa_2 samples were combined together and used for assembly.

Protein-coding sequences (CDSs) were predicted for each contig by using Prodigal v2.6.3 with the metagenomic mode [27]. Deduced amino acid sequences of the predicted CDSs were subjected to homology searches against the KEGG prokaryotic protein database [28] and NCBI nr database to assign functional and taxonomic annotation by using the easy-search and easy-taxonomy modes of MMSeqs release 9 [29], respectively. For the KEGG annotation, top hits with identity ≥ 40%, e-value < 1e−5, and bit-score ≥ 70 were used for further analyses. Homologs of the cyanobacterial photoreceptor genes *ccaS* of *Synechocystis* sp. PCC 6803 (ABI83649.1) and *rcaE* of *Tolypothrix* sp. PCC 7601/1 (BAM83580.1) were identified by BLASTP with the following criteria: identity ≥ 50%, alignment length ≥ 300 amino acids, and e-value < 1e−10.

The 16S rRNA gene sequences within contigs were first detected by a BLASTN search against the SILVA132 SSURef database [30] with the following criteria: identity ≥ 80%, e-value < 1e−20, and aligned length ≥ 200 bp. These sequences were further aligned against the SILVA132 and taxonomically annotated by using SINA v1.4.0 [31] with the minimum similarity threshold of 0.9. In SILVA132, conventional class *Betaproteobacteria* has been reclassified as the order *Betaproteobacteriales* under the class *Gammaproteobacteria*. Here, we regard *Betaproteobacteriales* as its own class, *Betaproteobacteria*.

### Gene abundance analysis

The read pairs used for assembly were mapped against the generated contigs by bowtie2 v2.3.5.1 [32]. Read abundances of the genes were calculated as the mean depth of the mapped reads within the gene-coding regions (i.e., [sum of per base read depth] / [gene length]). Calculation was conducted by using bedcov utility of samtools v1.9 [33]. Abundances of CDSs and 16S rRNA genes were summarized based on their assigned features (KEGG orthology identifiers and family-level taxonomy, respectively). Features that were mapped with read abundance < 10 in any sample or were detected in fewer than three samples were removed. The read abundance value for each feature was converted to a probability based on Monte Carlo sampling from the Dirichlet distribution and was subjected to a centered log-ratio transformation by using ALDEx2 v1.20.0 to analyze compositional read count data [34]. The median values of the centered log-ratio-transformed values from 128 Monte Carlo instances were used for the principal component analysis (PCA) and differential abundance analyses between the Polar and HMA samples. Significance of differential abundance was tested using Wilcoxon’s rank-sum tests followed by Benjamini–Hochberg correction.

### Construction of the metagenomic-assembled genomes

Contigs were clustered into bins by MetaWRAP [35] binning pipeline in combination with MetaBAT2 v2.15 [36], MaxBin v2.27 [37], and CONCOCT v1.1.0 [38]. Obtained bins were refined through the MetaWRAP bin_refinement and reassemble_bins pipelines with SPAdes v3.14.1 [39]. Completeness and contamination rates of the refined bins were calculated based on the presence/absence of 104 single-copy genes conserved in *Bacteria* by using CheckM v1.0.12 [40]. Bins achieving completeness of ≥ 30% and contamination rates of < 10% were treated as metagenome-assembled genomes (MAGs). The taxonomic affiliation of MAGs was assessed by using GTDB-Tk v1.4.0 [41]. Pairwise average nucleotide identity (ANI) between the MAGs was calculated with FastANI v1.1 [42].

### Phylogenetic analysis of Cyanobacteria

Phylogenetic trees were constructed based on the deduced protein sequences identified in the MAGs and the reference genomes by using PhyloPhlAn v3.0.58 [43] with RAxML-NG v0.9.0 [44] and the LG + I + G4 + F model. In addition, near full-length 16S rRNA genes that were sequenced previously [11] were aligned by using ARB software [45] and were used for phylogenetic tree construction with RAxML-NG with the GTR + I + G4 model. Node support was estimated by 1000 bootstrap resamplings.

### Spectral analysis of cryoconite

Approximately 100-200 mg of each cryoconite sample was re-suspended in 1 ml of 100 mM potassium phosphate buffer, pH 7.0, then the sample was disrupted with an equal amount of zirconia/silica beads (ϕ100 mm, BioSpec Products, Bartlesville, OK, USA) for 3 min at 30 Hz using the TissueLyser II (Qiagen, Hilden, Germany). The homogenates were incubated with rotation for 30 min at room temperature in the dark in the presence of 2% (v/v) Triton X-100 and then were centrifuged at 15,000×*g* for 5 min. Low-temperature fluorescence emission spectra of the dissociated phycobiliproteins within the supernatant were obtained at 540 nm excitation at 77K with a fluorescence spectrometer (Model FP8300; JASCO, Tokyo, Japan) equipped with a liquid nitrogen cooling unit (Model PMU-830; JASCO).

## Results

### Bacterial taxonomic composition of cryoconite

We sequenced metagenomes of 19 of 21 cryoconite samples (Additional file 1: Table S1, two samples [YlSt2 and KirS2] were used only for the spectral analysis), and obtained in total 7.3M contigs with a total length of 9.9 gigabases assembled from 776.7 million high-quality read pairs (Additional file 1: Tables S1 and S2).

**Fig. 1:**
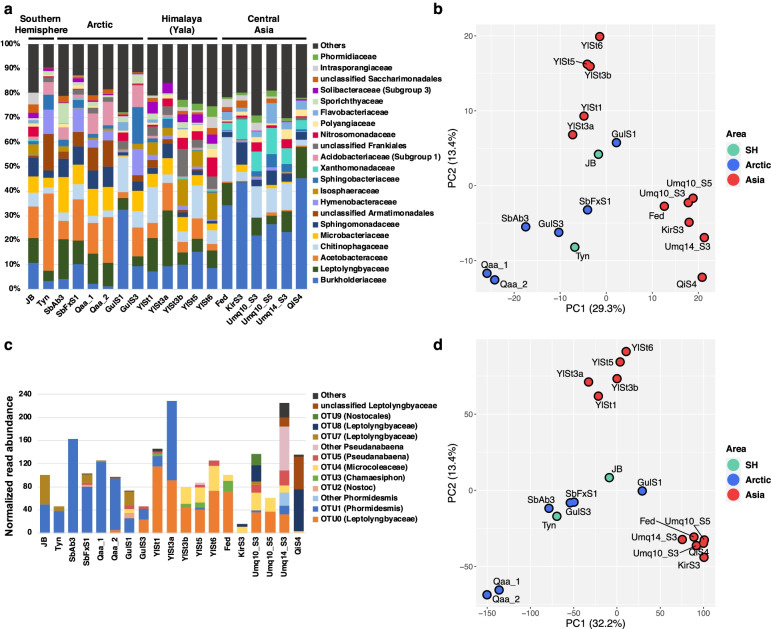
Comparison of bacterial community structures among cryoconite samples. **a** Bacterial taxonomic composition (mainly classified at the family level). **b** PCA based on the 16S rRNA gene abundance of bacterial families. SH, Southern Hemisphere. **c** Abundance and taxonomic affiliation of cyanobacterial 16S rRNA genes. Abundance was normalized to the total read abundance of 16S rRNA genes in each sample (adjusted to 1,000,000). **d** Principal component analysis based on the read abundance of KEGG-annotated genes

Diverse bacterial taxa were identified by analyzing the 16S rRNA genes within contigs (Fig. 1a and Additional file 2: Fig. S1 for family and class level, respectively), whereas no archaeal 16S rRNA genes were identified in this study. The PCA plot and hierarchical clustering based on the centered log-ratio-transformed read abundance of bacterial families indicated that the bacterial communities of some HMA samples (Fedchenko [Fed] in Pamir, Grigoriev [Kir: KirS3] in Tien Shan, Urumqi [Umq: Umq10_S3, Umq10_S5, and Umq14_S3] in Tien Shan, and Qiyi [QiS4] in Qilian Shan) were distinctly clustered from those of the Yala Glacier in the Himalayas (Yl: YlSt1, YlSt3, YlSt5, and YlSt6) and the non-Asian glaciers in this study (Fig. 1b and Additional file 2: Figure S2). Based on these results, we designated the samples Fed, KirS3, Umq (Umq10_S3, Umq10_S5, and Umq14_S3), and QiS4 as central Asian cryoconite and those from non-Asian regions as Polar cryoconite. Because the Patagonian sample (Tyn) was clustered with the Polar samples, we included Tyn in the Polar group.

Clear differences were observed in the composition of proteobacterial families between the Polar and HMA (especially, central Asian) cryoconite. *Burkholderiaceae* (*Betaproteobacteria*) was predominant in the central Asian cryoconite, accounting for 21.9–45.2% of the total 16S rRNA gene reads. Polar cryoconite also contained *Burkholderiaceae* but at a lower percentage than the central Asian cryoconite (Fig. 1a). In contrast, *Acetobacteraceae* (*Alphaproteobacteria*) was more abundant in the Polar cryoconite (2.55–31.5%) but represented < 1% of the gene reads in most of the central Asian samples (Fig. 1a). Differential analysis between the Polar and HMA (central Asian and Yl) samples indicated that *Acetobacteraceae* was significantly different between these two groups (Additional file 1: Table S3). In addition, strong positive and negative Pearson correlation values were seen between the first principal component (PC1) scores and the read abundance of *Burkholderiaceae* and *Acetobacteraceae*, respectively (Additional file 1: Table S3), suggesting that the relative abundance of these families contributed, in part, to the separation of central Asian cryoconite from Polar cryoconite along the PC1 axis (Fig 1b).

*Cyanobacteria* accounted for 1.5–23% (mean, 11%) of the total 16S rRNA gene reads in the samples (Additional file 2: Fig. S1). 16S rRNA gene fragments of cyanobacterial contigs were further associated with operational taxonomic units (OTUs), as identified previously by Segawa et al. [11], with 98% sequence identity. We excluded sequences of OTU6 from the OTU reference because we found that OTU6 was not assigned to *Cyanobacteria* based on the current SILVA classification.

Both the overall structure of the bacterial communities and the composition of the cyanobacterial lineages differed between the Polar and HMA cryoconite (Fig. 1c). In the Polar cryoconite, most of the cyanobacterial reads were affiliated with OTU1 in Segawa et al. [11] (mean, 72.2%): OTU1 is represented by *Phormidesmis priestleyi* (*Leptolyngbyaceae*), which is one of the most prevalent cyanobacterial species in the Polar regions [11, 46]. In contrast, OTU1 was less prevalent in the HMA cryoconite (mean, 2.42%), and OTU0, which is affiliated with the uncultured *Leptolyngbyaceae* lineage (Additional file 2: Fig. S3), was abundant (mean, 41.1%). It is notable that the HMA cryoconite contained several cyanobacterial lineages such as OTU0 and OTU8 (*Leptolyngbyaceae*), OTU3 (*Chamaesiphon*), OTU4 (*Microcoleaceae*), and OTU5 (*Pseudanabaena*), whereas the Polar cryoconite was almost solely dominated by OTU1 (Fig. 1c). These results are in agreement with our previous clone library-based observations [11].

### Profile of functional genes involved in essential metabolism in cryoconite

Similar to the 16S rRNA gene analysis, central Asian, Himalayan (Yl), and Polar cryoconite were clearly separated on the PCA plot based on the read abundance of KEGG-annotated prokaryotic functional genes (Fig. 1d and Additional file 2: Fig. S2). We then inspected the relative abundance and taxonomic affiliation of genes related to central metabolism in each sample.

### Carbon and nitrogen incorporation

For CO_2_ fixation, genes encoding key enzymes for the reductive pentose phosphate cycle, ribulose-bisphosphate carboxylase (*rbcLS*) and phosphoribulokinase (*prkB*), were consistently found in all samples (Additional file 2: Fig. S4). However, the presence of genes encoding key enzymes for other CO_2_ fixation pathways, such as acetyl-CoA decarbonylase/synthase in the reductive acetyl-CoA pathway and ATP-citrate lyase in the reductive citrate cycle, was negligible. Most *rbcL* and *prkB* homologs were affiliated with *Cyanobacteria*, *Betaproteobacteria*, and *Alphaproteobacteria*. Consistent with the taxonomic composition assessed by 16S rRNA genes, *rbcL* of *Betaproteobacteria* was abundant in the central Asian samples, whereas that of *Alphaproteobacteria* was more abundant in the Polar and Yl samples (Additional file 2: Fig. S4).

**Fig. 2:**
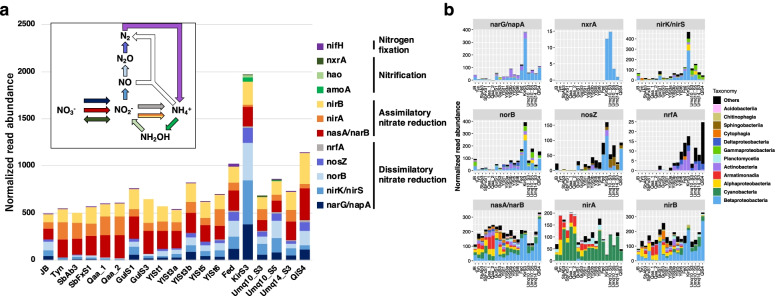
Abundance of the genes involved in nitrogen metabolism. Abundance was normalized to the total read abundance of KEGG-annotated genes in each sample (adjusted to 1,000,000). **a** Abundance of the key genes encoding key enzymes and/or subunits for nitrogen metabolism. Inset shows a schematic of inorganic nitrogen metabolism. Arrow colors corresponded to the colors of the responsible genes. The genes responsible for anaerobic ammonia oxidation, indicated by the white arrow, were not detected in this study. **b** Taxonomic composition of the genes involved in nitrate/nitrite reduction and oxidation

The genes responsible for nitrate assimilation (assimilatory nitrate reductase, *nasAB* and *narB*, and assimilatory nitrite reductase, *nirA*) were detected in all samples with comparable relative abundance (Fig. 2a). Similarly, genes encoding NADH-dependent nitrite reductase (*nirBD*) were detected. Although NirBD is classified as a dissimilatory nitrite reductase in KEGG, the activity of NirBD has not been directly linked to the respiratory chain: NirBD has been shown to be involved in nitrite assimilation and the maintenance of the redox balance by regenerating NAD^+^ [47–49]. We therefore regarded NirBD as the assimilatory nitrite reductase in this study. The majority of *nasA* (encoding catalytic subunit of NasAB), *narB*, and *nirB* (encoding large subunit of NirBD) homologs were affiliated with those of *Betaproteobacteria* and *Cyanobacteria* in the central Asian cryoconite, whereas *Alphaproteobacteria* and *Armatimonadia* were major bacteria carrying these genes in the Polar and Yl samples (Fig. 2b).

A marker gene for nitrogen fixation (*nifH*) was detected in 14 of 19 cryoconite samples, with SbAb3, GulS3, YlSt1, KirS3, and Umq10_S3 being the exceptions, although its relative read abundance was on average 100 times lower than those responsible for assimilatory nitrate reductase *nasAB*/*narB* (Fig. 2a; *p* < 0.001 by Wilcoxon signed-rank test).

### Energy conservation

The genes encoding key enzymes for aerobic respiration and fermentative metabolism (e.g., alcohol dehydrogenase, lactate dehydrogenase, and acetate kinase) were consistently detected from diverse bacterial classes (Additional file 2: Fig. S5 and S6), suggesting that these fundamental energy-producing mechanisms are conserved among the investigated cryoconite consortia. In contrast to the aerobic respiration, fermentation, and assimilatory nitrate reduction, significant regional differences were observed in the abundance of genes related to dissimilatory nitrate reduction, i.e., denitrification and dissimilatory nitrate reduction to ammonium. The genes encoding dissimilatory nitrate reductase (*narGHI*), nitrite reductase (*nrfA*, *nirK*, and *nirS*), nitric oxide reductase (*norBC*), and nitrous oxide reductase (*nosZ*), mainly affiliated with *Betaproteobacteria*, were significantly more abundant in the HMA cryoconite, especially central Asian cryoconite, than in the Polar samples (FDR < 0.1; Fig. 2 and Additional file 1: Table S4). Indeed, the read abundance of the genes for dissimilatory nitrate reduction largely contributed to the separation between the central Asian and Polar samples along the PC1 axis in Fig. 1d (Additional file 1: Table S4).

The genes responsible for ammonia oxidation (ammonia monooxygenase, *amoABC*, and hydroxylamine dehydrogenase, *hao*) and nitrite oxidation (nitrite oxidoreductase, *nxrAB*) were detected in some of the central Asian samples (KirS3, Umq10_S3, Umq10_S5, and Umq14_S3) but to a lesser extent than those for dissimilatory nitrate reduction (Fig. 2 and Additional file 1: Table S4). The genes for anaerobic ammonium oxidation (e.g., hydrazine synthase) were not detected in any sample.

A gene *dsrA*, encoding alpha subunit of dissimilatory sulfite reductase and responsible for dissimilatory sulfate reduction, was detected in the JB, YlSt6, and Fed, but its relative read abundance was ~ 25 times lower than that of *narG* in these samples and was almost negligible.

In the all samples, the genes encoding the components of Ni-Fe hydrogenase complexes were detected. Relative read abundance of genes indicated that H_2_ oxidizing Hya (HyaAB) and bidirectional, NAD^+^-dependent Hox (HoxEFHUY) were the predominant hydrogenases (Additional file 2: Fig. S7). Most of these hydrogenase genes did not show the significant differences between Polar and HMA regions, while *hoxH*, which encoding the large subunit of Hox hydrogenase, was relatively abundant in HMA samples than Polar ones (FDR < 0.1). In addition to the Hya and Hox, the genes for Ech and Hnd hydrogenases were detected mainly in HMA samples with relatively low abundance than those for Hya and Hox (Additional file 2: Fig. S7).

### Photosystem-related genes

**Fig. 3:**
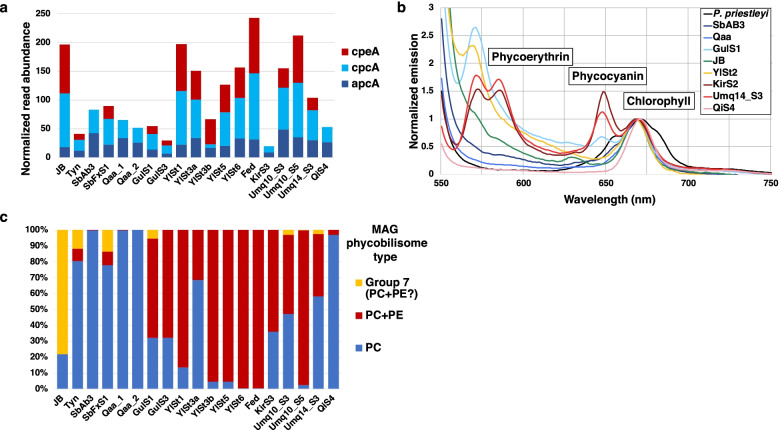
Profile of phycobiliprotein in the cryoconite samples. **a** Normalized abundance of the genes encoding alpha subunit of allophycocyanin (*apcA*), phycocyanin (*cpcA*), and phycoerythrin (*cpeA*). **b** Low-temperature fluorescence emission of the dissociated phycobiliproteins of the cryoconite samples and monoculture of *Phormidesmis priestleyi *at 540 nm excitation that preferentially excites phycoerythrin. **c** Composition of cyanobacterial lineages that possess phycocyanin only (PC) or both phycocyanin and phycoerythrin (PC + PE) based on the reconstructed MAGs and their relative read abundance (see Table S7 and Fig. S7). Because presence/absence of PE in the Group 7 MAGs was not identified in this study, the composition of Group 7 lineages was indicated separately

The genes encoding the components of the light-harvesting proteins and photosystem reaction center, as well as the enzymes for chlorophyll/bacteriochlorophyll *a* synthesis of the cyanobacterial oxygenic and proteobacterial anoxygenic phototrophs were detected in all samples (Additional file 2: Fig. S8). In *Cyanobacteria*, a protein supercomplex called phycobilisome is responsible for harvesting and transferring light energy to the photosystem [50]. In general, the phycobilisome consists of bilin chromophore-bound phycobiliproteins that absorb the red light part of the spectrum: allophycocyanin (APC, λ_max_ = 650 nm) and phycocyanin (PC, λ_max_ = 620 nm) [50]. Some strains can use green light for photosynthesis using phycoerythrin (PE, λ_max_ = 490-550 nm) [50]. In our cryoconite samples, genes encoding the component of APC and PC (i.e., *apcA* and *cpcA*, which encode the alpha subunit of APC and PC, respectively) were consistently detected in all samples, whereas those that encode PE were not detected in Qaa, SbAb3, KirS3, and QiS4 samples (Fig. 3a).

In addition to the metagenomic survey, we measured low-temperature fluorescence emission spectra of the frozen-stored cryoconite samples to clarify the in situ expression of phycobiliproteins. Emission peaks for PE around 570–580 nm were observed in the cryoconite collected at GulS1, Umq14_S3, KirS2, and YlSt2 (Fig. 3b; peaks for PC were also observed in GulS1, Umq14_S3, and KirS2), demonstrating that PE was expressed in cryoconite from Umq, Kir, and Yl in the HMA region and Gul in Alaska. A unialgal culture of *P*. *priestleyi* exhibited an emission peak for PC but not for PE, consistent with the absence of the PE genes in the *P*. *priestleyi*-dominated cryoconite in the Arctic glaciers (Qaa and SbAb3) (Fig. 3). On the other hands, we did not observe clear emission peaks for phycobiliproteins in SbAb3, Qaa, JB, YlSt2, and QiS4, suggesting that the amounts of PC and PE were below the detection limit in these samples.

### Genes for psychrotolerance

Various bacterial metabolisms are involved in mitigating cold and osmotic stress in ice environments [46, 51]. We found the genes for cold/heat-shock proteins (molecular chaperones), osmoprotectant biosynthesis and uptake, and exopolysaccharide export in a variety of bacterial lineages in the cryoconite consortia among the regions (Additional file 2: Fig. S9). Exopolysaccharides are important not only for psychrotolerance but also for the assemblage of cryoconite consortia and nutrients for heterotrophs [52]. In our metagenomes, the homologs of *wza/kspD*, which encode the exporter of polysaccharides, were mainly affiliated with *Cyanobacteria* (6.6–57.2% of relative read abundance in each sample; mean, 30.5%), *Betaproteobacteria* (0.66–47.5%; mean, 17.5%), and *Alphaproteobacteria* (3.0–35.5%; mean, 16.5%), implying that these bacterial lineages play roles not only in processing the central metabolisms described above but also in physically maintaining cryoconite community by secreting polysaccharides.

### Construction of metagenome-assembled genomes (MAGs) of Cyanobacteria

**Fig. 4:**
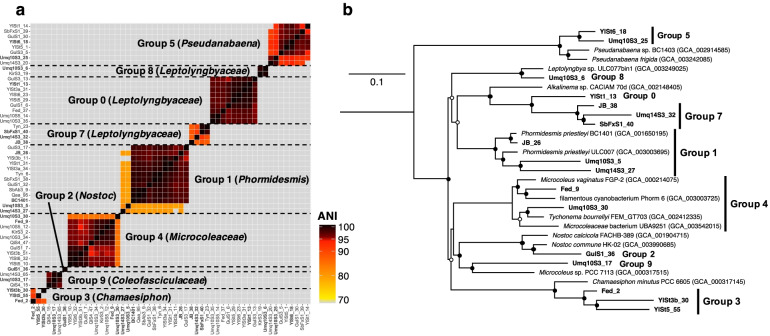
Phylogeny of metagenome-assembled genomes (MAGs) of the cyanobacterial lineages observed in the cryoconite samples. **a** Clustering of the MAGs based on the pairwise average nucleotide identity (ANI). MAG IDs indicated in bold were used for phylogenetic analysis in **b**. BC1401: *Phormidesmis priestleyi* BC1401. **b** Phylogenetic positions of the MAGs. Bootstrap confidence values of ≥ 50% (open circles) and ≥ 70% (filled circles) are indicated. *Gloeobacter violaceus* PCC 7421 (GCA_000011385) was used as the outgroup

In total, 52 cyanobacterial MAGs were retrieved from all assemblies (Additional file 1: Table S5) with mean completeness and a contamination rate of 81.37% and 0.63%, respectively (Additional file 1: Table S6). These cyanobacterial MAGs and the draft genome of *Phormidesmis priestleyi* BC1401 (GCA_001650195), derived from a cyanobacterial isolate from cryoconite on the Greenland Ice Sheet [46], were clustered into nine MAG groups with ≥ 70% ANI values (Fig. 4a). Most of the MAGs did not contain 16S rRNA genes, likely due to the difficulty in assembling rRNA genes [53, 54] (Additional file 1: Table S6). We were, however, able to putatively number these nine MAG groups according to their corresponding OTU numbers (e.g., Group 0 corresponds to OTU0) by comparing the phylogenetic positions of the MAGs (Fig. 4b) with the 16S rRNA gene OTUs that we identified previously [11] (Additional file 2: Fig. S3).

Based on the ANI threshold of 95% for the species delineation [42], our cyanobacterial MAGs comprised 17 species-level lineages. Group 1 MAGs from the Polar and Yl samples were identified as *P. priestleyi* because they showed ANI values of ≥ 95% with the draft genome of *P. priestleyi* BC1401. In contrast, two other Group 1 MAGs from Umq (Umq10S3_5 and Umq14S3_27) had ANI of < 90% relative to the other Group 1 MAGs and *P. priestleyi* BC1401 (Fig. 4a), suggesting that these MAGs in Umq cryoconite originated from different cyanobacterial species than the Polar-dominant *P. priestleyi*. We estimate the composition of these 17 species-level lineages based on the read abundance mapped on the MAGs (Additional file 2: Fig. S10) and found that it was consistent with what was assessed based on 16S rRNA genes (Fig. 1c).

### Metabolic potential of glacial Cyanobacteria assessed by the MAG analysis

As we detected different read abundance levels for the nitrogen metabolism- and photosystem-related genes between the HMA and Polar cryoconite metagenomes, we analyzed the presence of these genes in the cyanobacterial MAGs. At least one MAG among nine MAG groups possessed genes encoding a nitrate/nitrite transporter (*nrtABC*) and assimilatory nitrate/nitrite reductase (*narB*/*nirA*) as well as ammonium transporters (*amt*), urea transporter (*urtABCDE*), and urease (*ureABC*). In contrast, the gene set for nitrogen fixation (*nif*) was not found in any of the MAGs except for GulS1_36 (Group 2; *Nostoc*). At least one MAG in Groups 0, 3, 4, 7, and 8, which were recovered mainly from the HMA cryoconite (Additional file 2: Fig. S10), contained two gene sets for the nitrite reductases (*nirA* and *nirBD*)*.* However, the genome of *P. priestleyi* BC1401 and the related MAGs in Group 1 (with ≥95% ANI values) possessed *nirA* and *nirD* (encoding small subunit of NirBD) but lacked *nirB*.

The presence of photosystem-related genes also differed among the MAGs. Group 1 MAGs (*Phormidesmis*) contained the gene sets for APC and PC but not for PE. The PE genes were detected in the MAGs from four MAG groups: Groups 0, 3, 4, and 5 (Additional file 1: Table S7). Composition of cyanobacterial lineages based on the MAGs indicated that most of the HMA cryoconite were dominated by lineages that possess both PC and PE, in contrast to the Arctic samples where the PE-absent *P. priestleyi* was predominant (Fig. 3c and Additional file 2: Fig. S10).

Possession of APC, PC, and PE can influence the ability of *Cyanobacteria* to use different wavelengths of light. Certain cyanobacterial species are capable of changing the combination of phycobiliproteins in response to the available wavelength of light, known as chromatic acclimation (CA) [55]. There are two well-characterized CA systems, CA2 (which regulates the expression of PE alone) and CA3 (which regulates the composition of PC and PE), which are regulated by the photoreceptors CcaS and RcaE, respectively [56, 57]. In the cyanobacterial MAGs, *ccaS* homologs were detected in two Group 3 MAGs from Yl (YlSt3b_30 and YlSt5_55; *Chamaesiphon*), whereas *rcaE* homologs were detected in two Group 7 MAGs (JB_38 and Umq14S3_32; *Leptolyngbyaceae*) and two Group 5 MAGs (YlSt5_1 and YlSt6_18; *Pseudanabaena*) (Additional file 1: Table S7). In addition, although not retrieved in the MAGs, we found a homolog of *ccaS* (99.7% sequence identity with those detected in YlSt3b_30 and YlSt5_55) and *rcaE* (99.1% sequence identity with that detected in Umq14S3_32) in the contigs of Fed and Umq10_S3, respectively. Thus, all of *ccaS* and *rcaE* homologs, except one encoded in MAG JB_38 from JB, were detected from the HMA samples. Given that *Cyanobacteria*-conserved APC and PC genes were absent from some MAGs (Additional file 1: Table S7) because of the incompleteness of the genomes, the inconsistency between the absence of PE and the presence of *rcaE* homologs in Group 7 MAGs might also be attributed to their incompleteness (Additional file 1: Table S7).

## Discussion

**Fig. 5:**
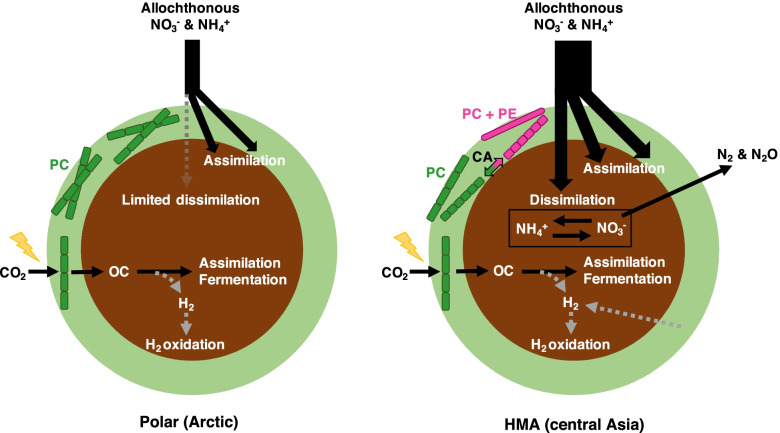
Hypothesized metabolism of cryoconite bacterial consortia of Polar (Arctic) and High-Mountain Asia (HMA, central Asia) glaciers. Outer light-green and inner brown parts indicate surface oxic layers dominated by *Cyanobacteria* and inner anaerobic/microaerobic cores of cryoconite granules, respectively. OC: organic compounds; PC: phycocyanin; PE: phycoerythrin

Our metagenomic investigation successfully revealed regionally distinct taxonomic assemblages and their associated metabolic potential of glacier surface cryoconite. Notably, the greater abundance of the genes for denitrification in the HMA samples relative to that in the Polar counterparts indicates that bacteria that reduce nitrate for respiration thrive in HMA cryoconite but not in Polar settings (Fig. 5). Indeed, active denitrification was observed in Umq cryoconite by the quantification of transcripts of the denitrification genes and isotopic analyses [7, 58]. Denitrification is generally found to occur in the anaerobic/microaerobic conditions. Previous studies have reported the presence of anoxic cores inside cryoconite granules from Greenland Ice Sheet and Umq [58, 59]; therefore, anoxia alone cannot explain the distinct abundance of denitrifiers between Polar and HMA cryoconite. One of the most plausible factors influencing the occurrence of denitrifiers is a higher concentration of substrates for denitrification, i.e., nitrate and dissolved organic carbon (DOC) [60], in glacial meltwater in HMA glaciers relative to that found in Polar environments [61–63]. Elevated concentrations of nitrate and DOC can be attributed to anthropogenic activities and dust derived from arid inland regions in central Asia that affects the composition of precipitation [61, 62]. Furthermore, HMA glaciers, including those in our study, are typically characterized by steeper, narrower valley topography, and a greater exposed valley flank area, which increases the likelihood of debris fall and avalanches that might represent a source of denitrifiers and nutrients (including DOC and nitrate). A recent transcriptomic analysis demonstrated the high transcription of genes related to denitrification in cryoconite from the Forni Glacier in the Italian Alps [64], suggesting that denitrification activity may be high not only in HMA glaciers but also among a wider range of mid-latitude alpine glaciers. As denitrification ultimately reduces nitrate to nitrogenous gases, active denitrification in alpine glaciers would affect nitrogen flows not only within the local glacier ecosystem but also to its downstream environments.

In addition to the input from surrounding environments, nitrate can also be generated via nitrogen mineralization and nitrification, potentially supporting denitrifiers in cryoconite. Recent isotope and transcript analyses have shown nitrification activity in Umq cryoconite [7, 58], and our current study also detected *amoA* and *nxrAB* in both Umq and KirS3 (Tien Shan) samples. In the Tien Shan, deposition of nitrate and atmosphere-derived ammonium occurs at higher concentrations than in other regions [65]. Such high substrate concentrations may facilitate the proliferation of both denitrifying and nitrifying bacteria in glaciers from the Tien Shan region. We note that previous studies detected *amoA* in some Arctic and Antarctic cryoconite by using gene-specific PCR [66, 67]. Thus, ammonia-oxidizing prokaryotes may also be present in our Polar samples although their abundance may be low. Similarly, bacteria that possessed the machinery for nitrogen fixation were minor among all examined samples. This result, as well as findings from previous studies [58, 68], suggested that nitrogen fixation marginally contributes to nitrogen incorporation in the cryoconite, although it may be seasonally or conditionally active [69].

Besides dissimilatory nitrate reduction, fermentation and hydrogen metabolism are other major metabolisms that can generate energy under the anaerobic/microaerobic conditions. Our metagenomic data indicated that these metabolisms could occur among various bacterial classes and geographical regions. Recent transcriptomic study done with Forni Glacier cryoconite also suggests the active transcription of *hyaB*, which encodes the large subunit of H_2_ oxidizing hydrogenase Hya [64]. Given that *hyaB* was also detected in both Polar and HMA cryoconite in this study, H_2_ oxidation might be common for the energy conservation in cryoconite (Fig. 5). Interestingly, the cyanobacterial Hox hydrogenase genes were detected in the HMA samples (Additional file 2: Fig. S7), which was coded in the Group 8 MAGs. Therefore, H_2_ might be produced from this cyanobacterial lineage in the HMA cryoconite (Fig. 5). A physiological study on the cyanobacterium *Synechocystis* suggested that the Hox hydrogenase contributes to maintaining the redox balance when photosynthetic and respiratory pathways are downregulated under the prolonged dark condition [70]. Thus, Hox hydrogenase activity might support the survival of certain cyanobacterial lineages on the glacier surface where many factors (e.g., snow covering, low temperature) could inhibit their normal photosynthetic activity.

Within the HMA cryoconite sample set, Yala Glacier (Yl) was distinct from the central Asian sites, with respect to both taxonomic and functional gene composition (Fig. 1b, d), suggesting the presence of different controlling environmental factors. One plausible factor is the difference in climate: Yl, located in the southern slopes of the Himalaya range, is subject to the influence of the monsoon in contrast to the central Asian glaciers described here, which have a more continental, arid climate [61]. This climatic contrast, therefore, likely controls the amount and quality of dry and wet nutritional substrates supplied from the atmosphere. For instance, the supraglacial nitrate concentration is lower in Yl than the central Asian glaciers, including Umq [61], which might result in the relatively low read abundance of the genes responsible for denitrification (e.g., *nosZ*) in Yl than the central Asian samples (Fig. 2b and Additional file 1: Table S4). Furthermore, the sources of the wind-blown mineral dust found on glaciers in the Himalayas are different from those on the glaciers in central Asia [71]. Such environmental differences may collectively shape distinct cryoconite consortia between the monsoon-affected Himalayas (e.g., Yl) and central Asia, suggesting that geographical contrasts in cryoconite functionality may exist within HMA in response to regional climatology.

We further unveiled the genomic features of the various cyanobacterial lineages in the examined samples, including those without culture representatives (e.g., Group 0). All cyanobacterial MAG groups contained genes responsible for the assimilation of nitrate/nitrite, ammonium, and urea, suggesting that *Cyanobacteria* in cryoconite can use these nitrogen compounds like other known cyanobacterial species [72]. Interestingly, several cyanobacterial lineages predominant in the HMA cryoconite harbored two gene sets for assimilatory nitrite reductase: ferredoxin-dependent NirA and NADH-dependent NirBD. Indeed, cyanobacterial *nirB* was specifically detected from the HMA samples (Fig. 2b). The activity of NirA in cyanobacteria has been well documented; reduced ferredoxin from photosynthesis is used for the reduction of nitrite to ammonium [73]. In contrast, the physiological role of NirBD in cyanobacteria is not well known, although the expression of NirBD in *Microcoleus* sp. was detected in a freshwater benthic biofilm [74]. A study of *Escherichia coli* demonstrated that the nitrite reduction by NirBD provides an oxidative power to regenerate NAD^+^ in addition to ethanol and lactate fermentation, thereby promoting the synthesis of ATP [48]. Fermentation by *Cyanobacteria* in anoxic microbial mats was previously reported [75], and all MAG groups obtained in this study contained the genes important for fermentation such as d-lactate dehydrogenase, alcohol dehydrogenase, and acetate kinase. Taken together, these findings suggest that NirBD of *Cyanobacteria* may not only be involved in nitrite assimilation but also confer an oxidative power for NAD^+^ regeneration in addition to fermentation under anoxic conditions. *Cyanobacteria* capable of this reaction might have a selective advantage in nitrate- or nitrite-rich HMA cryoconite and, therefore, be more abundant in Asian than Polar cryoconite.

In addition to nitrite reductase genes, cyanobacterial genes for light-harvesting components in the phycobilisome were distributed differently between the Polar and HMA cryoconite, highlighted by the predominance of PE-absent *P. priestleyi* and PE-possessing lineages in the Arctic and HMA samples, respectively (Figs. 3a, c and 5). Such distinctive distribution of PE genes might reflect the different approaches of cyanobacterial lineages for the usage of phycobilisome: one of the most abundant proteins and accounts for up to ~50% of the total soluble protein in *Cyanobacteria* [76]. In Polar glaciers, the growth of cyanobacteria would be limited by the low availability of nutrients rather than that of light energy. Under this condition, *Cyanobacteria* possessing PE-absent, relatively low-cost phycobilisome (e.g., *P. priestleyi*) might be adaptive. This assumption is also suggested by the genome of *Leptolyngbya* sp. BC1307 from McMurdo Dry Valleys, Antarctica: this cyanobacterial strain completely lacks PE and CA components from its genome while its close relatives possess intact ones [77]. In contrast, HMA glaciers, especially those in central Asia where relatively high amounts of nutrients are available, could sustain more diverse cyanobacterial lineages and lead to severe light competition within a single cryoconite granule. Cyanobacterial lineages possessing PE and CA systems can mitigate the light competition by using a broader range of wavelength and therefore may thrive in HMA cryoconite [78]. Moreover, alpine glaciers located at lower latitudes are expected to be exposed to more short-wavelength irradiance than Polar regions due to their higher elevation and solar altitude, which could provide more opportunities for the PE-possessing *Cyanobacteria* to harvest energy.

The amounts of PC or PE were below the detection limit in some cryoconite samples (Fig. 3b). These observations might reflect the self-degradation of phycobilisomes that enable *Cyanobacteria* to re-allocate resources and avoid excessive photosynthesis when facing unfavorable conditions (e.g., nutrient limitation, low temperatures) [76, 79]. *Cyanobacteria* thus might utilize phycobilisomes not only for light antenna but also reservoir of substrates in the fluctuating glacier environments. The dynamics of phycobilisome production/degradation in cryoconite will be an important topic to be elucidated for the further understanding on the adaptation of *Cyanobacteria* to glacier environments.

## Conclusions

Our comparative metagenomic study revealed, for the first time, that the bacterial microbiomes of High-Mountain Asian glacier cryoconite are distinct from their more-commonly described Polar counterparts. High-Mountain Asian cryoconite was characterized by the predominance of *Betaproteobacteria* and multiple cyanobacterial lineages other than Polar-predominant *P. priestleyi*, a substantial potential for dissimilatory nitrate reduction, and the presence of phycoerythrin and CA systems in *Cyanobacteria*. Our results highlight environmental controls on cryoconite microbe assemblages, with enhanced potential of dissimilatory nitrate reduction and light harvesting in cryoconite on lower latitude, high elevation glaciers. Recognition of the role of these environmental controls on metabolic potential within glacier ecosystems is essential to better understand biodiversity, biogeochemical cycling, and forecast the functional and ecological transitions that will be driven by future climatic change and glacier decline.

## Supplementary Information

**Additional file 1: Table S1**. Information on sampling sites and sequencing of the cryoconite samples. **Table S2.** Assembly status of each metagenomic sample. **Table S3.** Centered log-ratio transformed read abundance of the top 20 most abundant bacterial families. **Table S4.** Centered log-ratio transformed read abundance of the genes involved in inorganic nitrogen metabolism. **Table S5.** Number of metagenome-assembled genomes (MAGs) obtained from each assembly. **Table S6.** Assembly status and taxonomic affiliation of the metagenome-assembled genomes (MAGs) of *Cyanobacteria*. **Table S7.** Number of genes encoding phycobiliproteins and photosensor homologs for chromatic acclimation in each cyanobacterial metagenome-assembled genome (MAG).

**Additional file 2: Figure S1.** Bacterial class composition based on 16S rRNA read abundance. **Figure S2.** Hierarchical clustering of cryoconite samples based on the abundance of 16S rRNA genes (upper) and KEGG-annotated genes (lower). **Figure S3.** Phylogenetic positions of 16S rRNA gene phylotypes of *Cyanobacteria*. **Figure S4.** Read abundance of the genes responsible for CO_2_ fixation. **Figure S5.** Read abundance of the genes responsible for aerobic respiration. **Figure S6.** Read abundance of the genes involved in the fermentation. **Figure S7.** Read abundance of the genes encoding the subunit of hydrogenases. **Figure S8.** Read abundance of the genes responsible for photosystems. **Figure S9.** Read abundance of the genes involved in the psychrotolerance. **Figure S10.** Composition of cyanobacterial lineages based on the read abundance of the metagenome-assembled genomes (MAGs).

## Data Availability

FASTQ files of shotgun metagenomic reads were deposited under the accession number PRJDB11497. Cyanobacterial MAGs have been uploaded in 10.6084/m9.figshare.14776452.

## Abbreviations

APC: Allophycocyanin
CA: Chromatic acclimation
DOC: Dissolved organic carbon
FDR: False discovery rate
HMA: High-Mountain Asia
KEGG: Kyoto Encyclopedia of Genes and Genomes
MAG: Metagenome-assembled genome
OTU: Operational taxonomic unit
PC: Phycocyanin
PE: Phycoerythrin
