# Supplementary material for: Metagenomics reveals global-scale contrasts in nitrogen cycling and cyanobacterial light-harvesting mechanisms in glacier cryoconite
Source: Microbiome. 2022 Mar 23;10:50. doi: 10.1186/s40168-022-01238-7 (PMC8941735; doi:10.1186/s40168-022-01238-7)
Supplement: Supplementary file 3 — Additional file 2: Figure S1. Bacterial class composition based on 16S rRNA read abundance. Figure S2. Hierarchical clustering of cryoconite samples based on the abundance of 16S rRNA genes (upper) and KEGG-annotated genes (lower). Figure S3. Phylogenetic positions of 16S rRNA gene phylotypes of Cyanobacteria. Figure S4. Read abundance of the genes responsible for CO2 fixation. Figure S5. Read abundance of the genes responsible for aerobic respiration. Figure S6. Read abundance of the genes involved in the fermentation. Figure S7. Read abundance of the genes encoding the subunit of hydrogenases. Figure S8. Read abundance of the genes responsible for photosystems. Figure S9. Read abundance of the genes involved in the psychrotolerance. Figure S10. Composition of cyanobacterial lineages based on the read abundance of the metagenome-assembled genomes (MAGs). [file 40168_2022_1238_MOESM3_ESM.pdf]

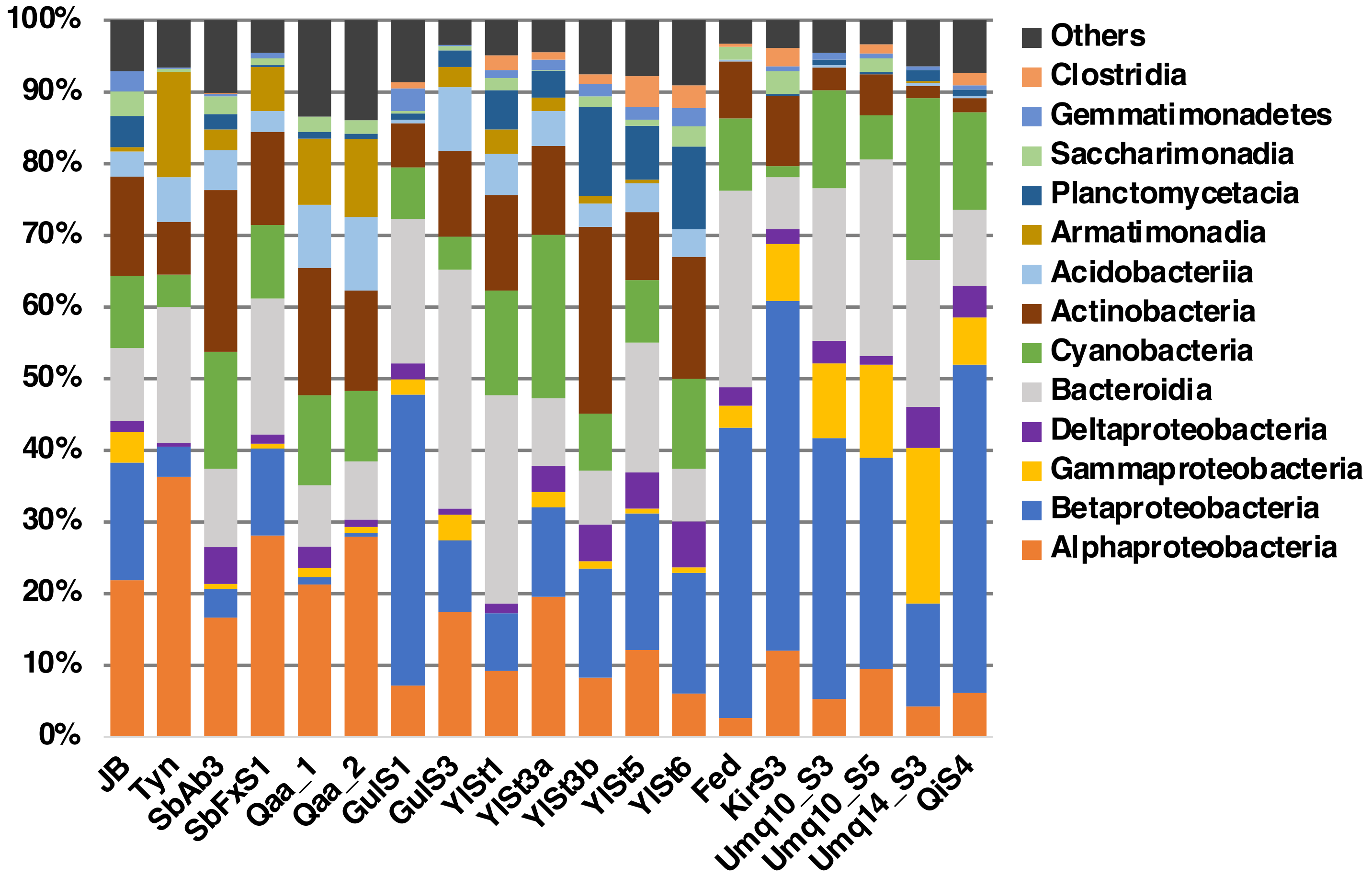

Figure S1. Bacterial class composition based on 16S rRNA read abundance. Taxonomy follows SILVA132 except for Betaproteobacteriales and Oxyphotobaceria; these are indicated as Betaproteobacteria and Cyanobacteria, respectively.

16S rRNA gene

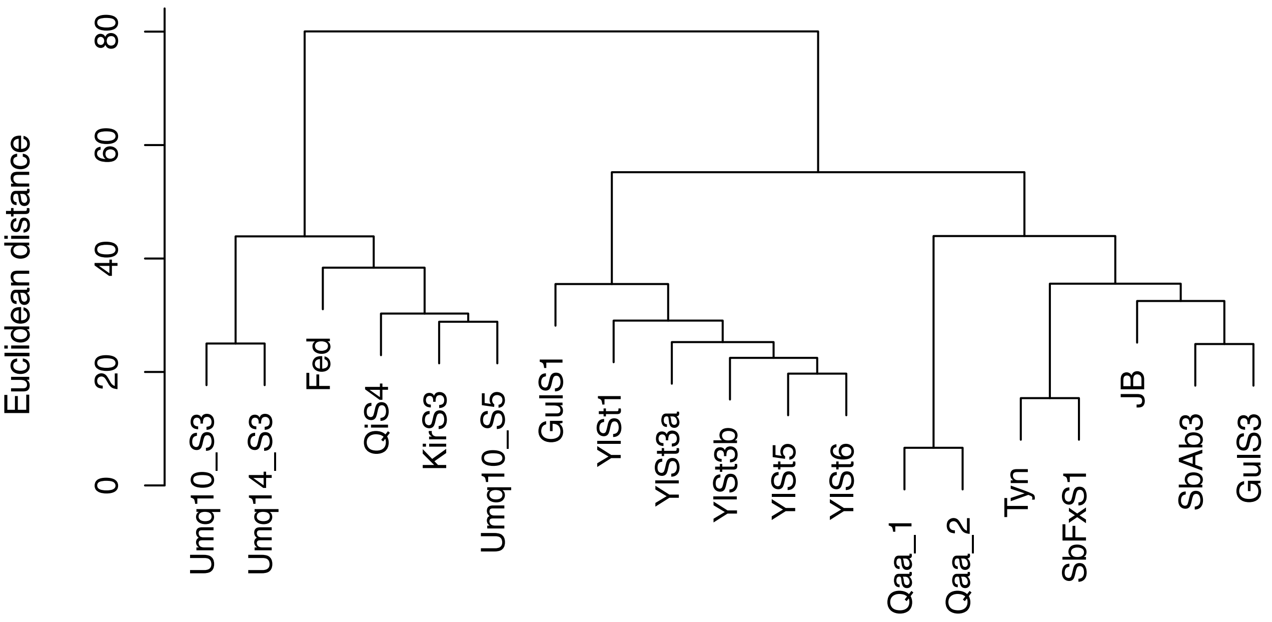

KEGG-annotated gene

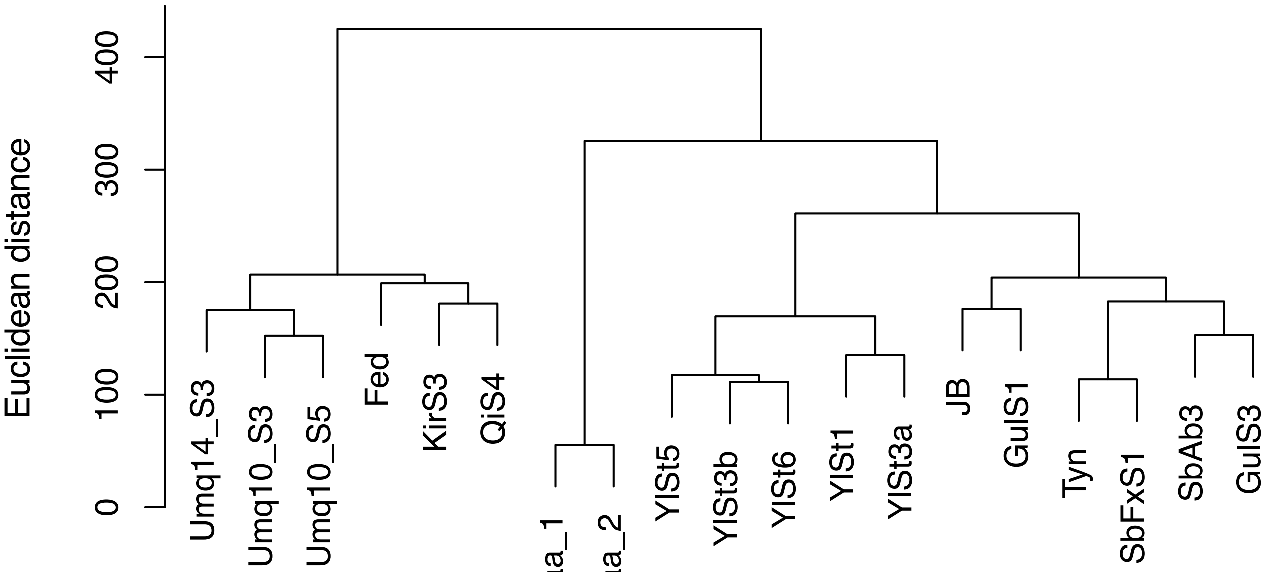

Figure S2. Hierarchical clustering of cryoconite samples based on the abundance of 16S rRNA genes (upper) and KEGG-annotated genes (lower).
Pairwise Euclidean distance between the samples was used for clustering with the Ward method. Calculations were carried out by using the *dist* and *hclust* functions in R software.

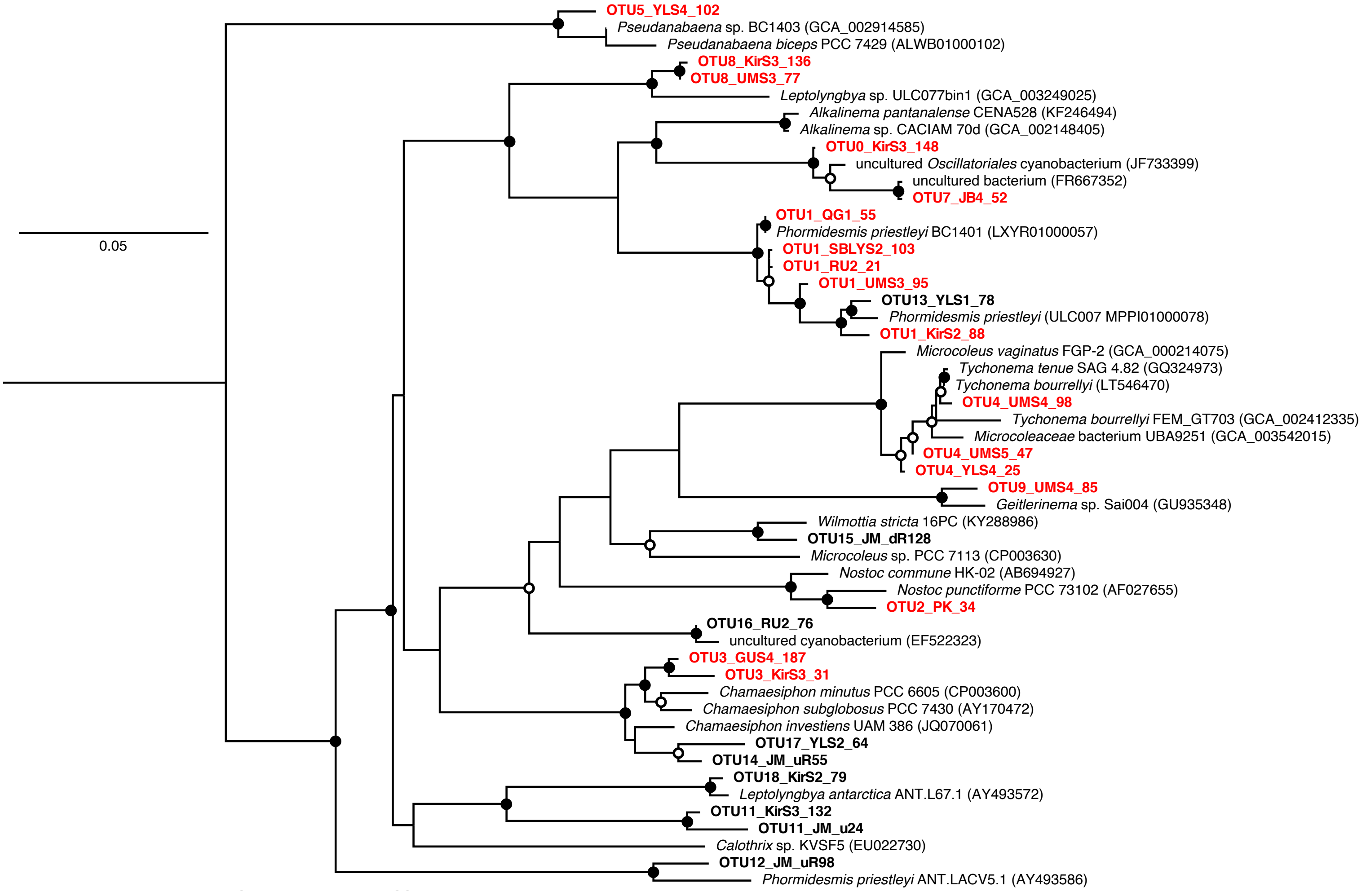

Figure S3. Phylogenetic positions of 16S rRNA gene phylotypes of *Cyanobacteria*. Sequences of the operational taxonomic units (OTUs; indicated in bold) were clone-sequenced from the cryoconite samples in Segawa et al. (2017). The OTU groups detected in the present study are shown in red. Bootstrap confidence values ≥50% (open circles) and ≥70% (filled circles) are indicated. *Gloeobacter violaceus* PCC 7421 (AF132790) was used as the outgroup.

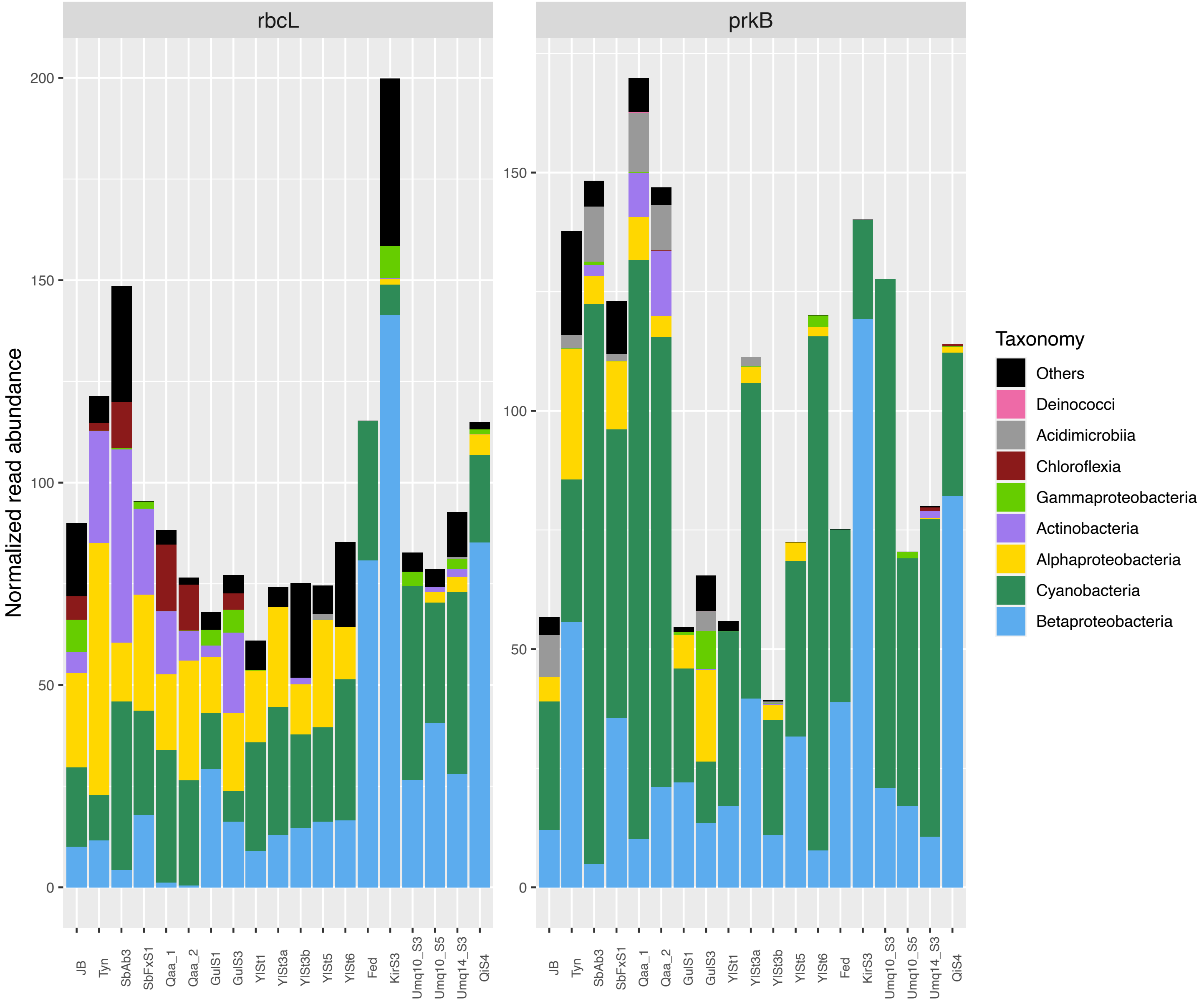

Figure S4. Read abundance of the genes responsible for CO_2 fixation.
Read abundance was normalized to the total read abundance of KEGG-annotated genes in each sample. *rbcL*: ribulose-bisphosphate carboxylase large subunit; *prkB*: phosphoribulokinase.

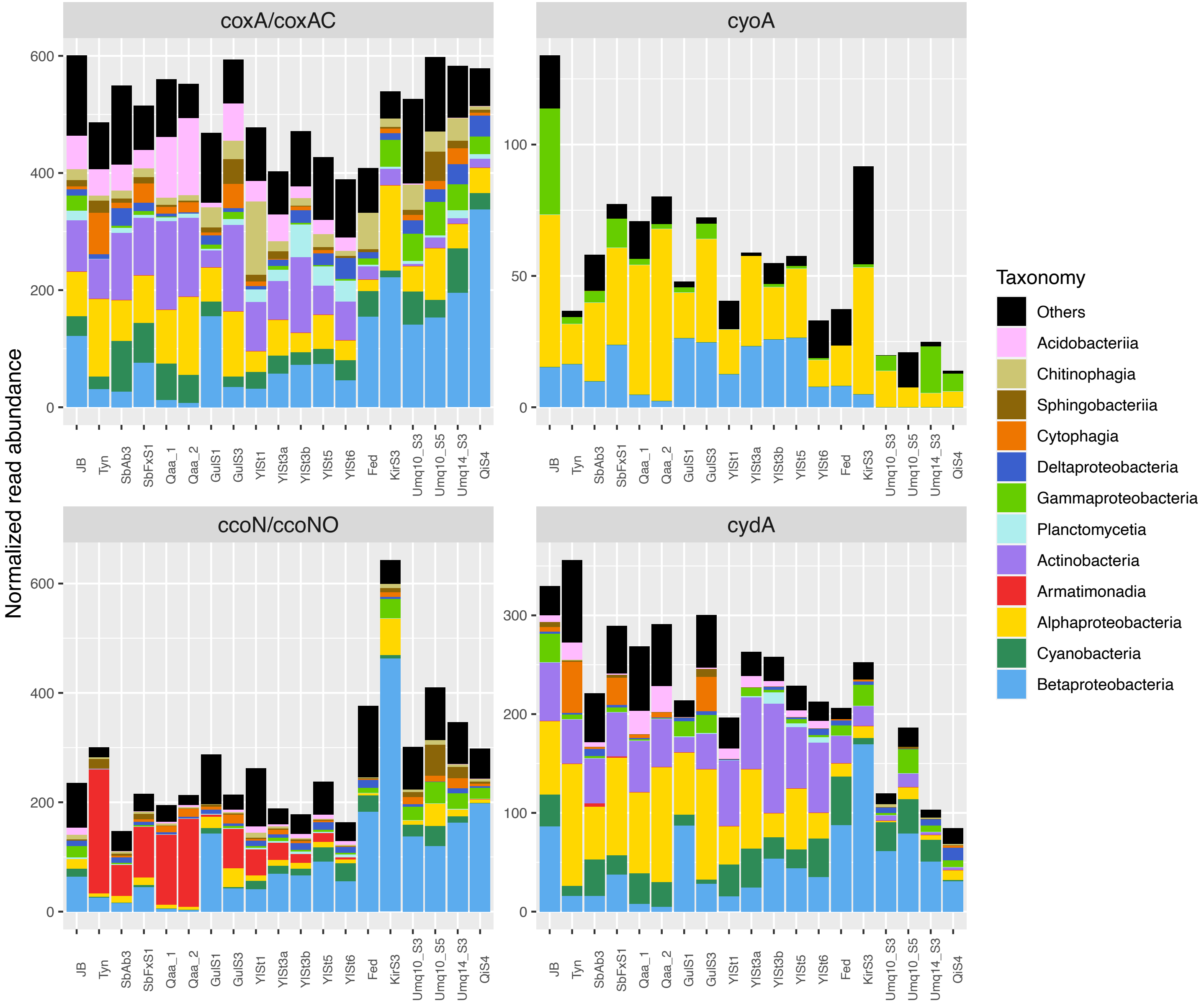

Figure S5. Read abundance of the genes responsible for aerobic respiration. Read abundance was normalized to the total read abundance of KEGG-annotated genes in each sample. *coxA/coxAC*: cytochrome *c* oxidase subunit I (+ III); *cyoA*: cytochrome *o* ubiquinol oxidase subunit II; *ccoN/ccoNO*: cytochrome *c* oxidase cbb3-type subunit I (+ II) *cydA*: cytochrome *bd* ubiquinol oxidase subunit I.

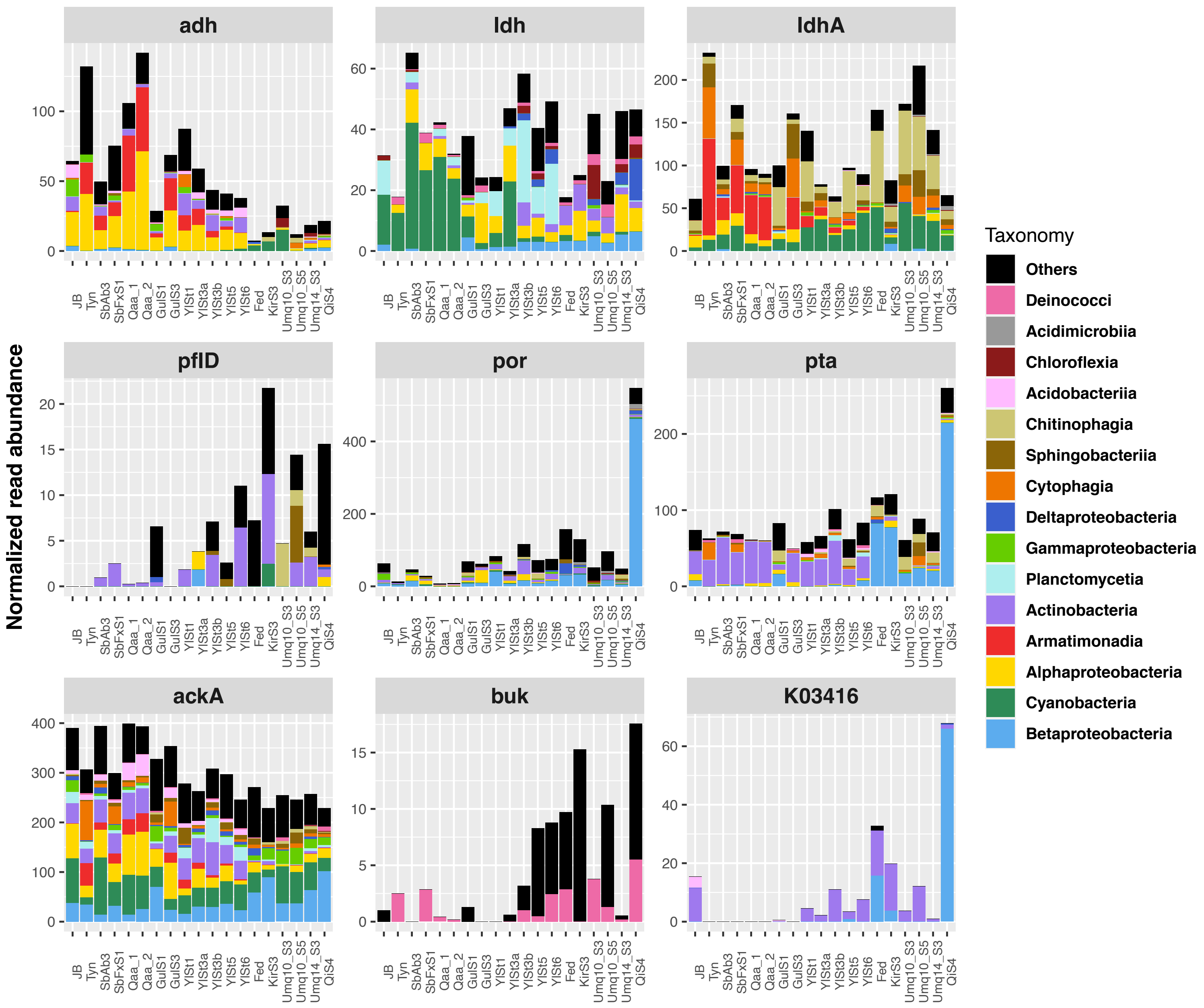

Figure S6. Read abundance of the genes involved in the fermentation.
Read abundance was normalized to the total read abundance of KEGG-annotated genes in each sample. *adh*: alcohol dehydrogenase (KEGG Orthology ID: K00001 and K00002); *ldh*: L-lactate dehydrogenase; *ldhA*: D-lactate dehydrogenase; *plfD*: formate C-acetyltrans -ferase; *por*: pyruvate-ferredoxin oxidoreductase; *pta*: phosphate acetyltransferase; *ackA*: acetate kinase; *buk*: butyrate kinase; K03416: methylmalonyl-CoA carboxyltransferase.

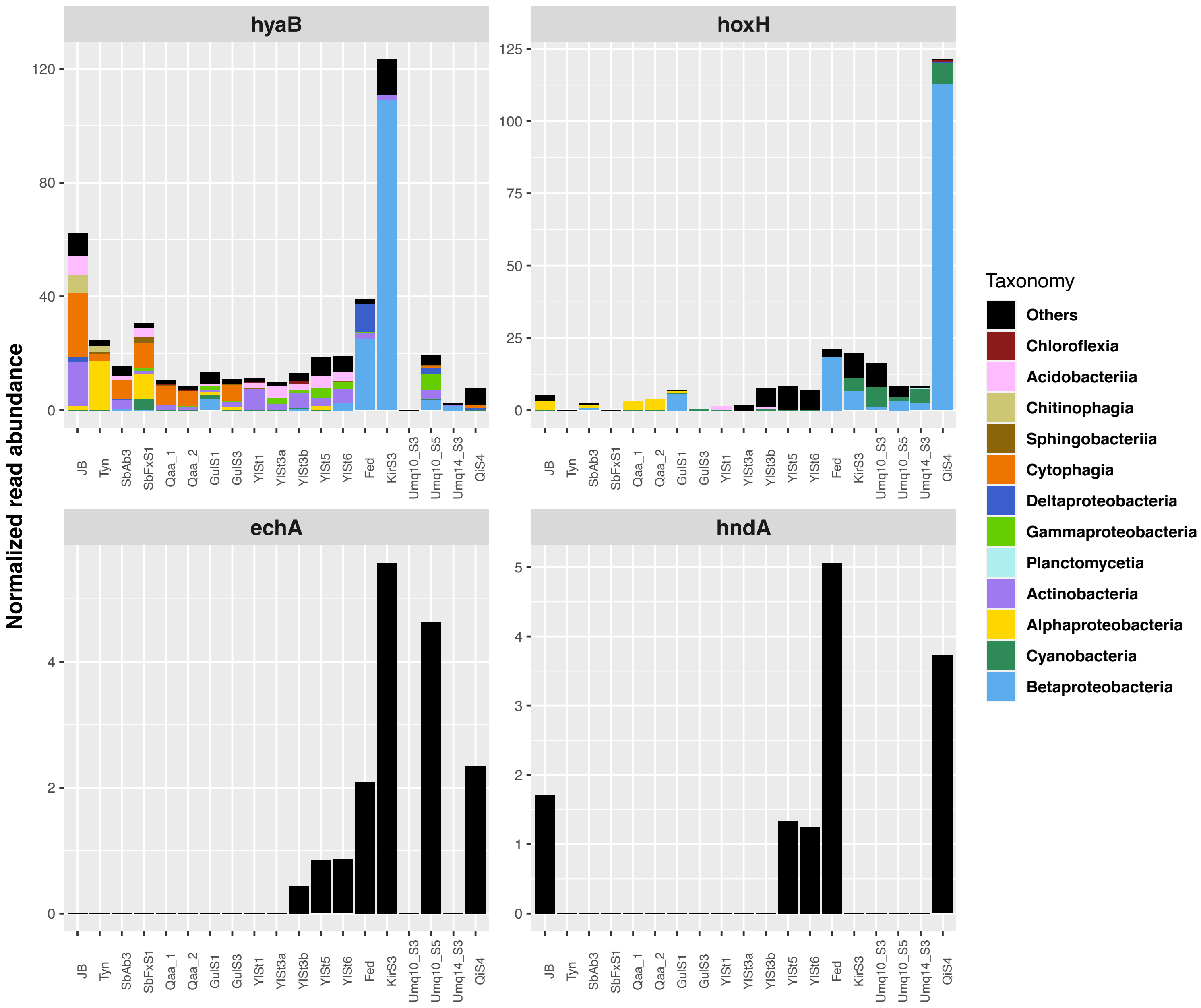

Figure S7. Read abundance of the genes encoding the subunit of hydrogenases
Read abundance was normalized to the total read abundance of KEGG-annotated genes in each sample. *hyaB*: Hya hydrogenase large subunit; *hoxH*: Hox hydrogenase large subunit; *echA*: Ech hydrogenase subunit A; *hndA*: Hnd hydrogenase subunit A.

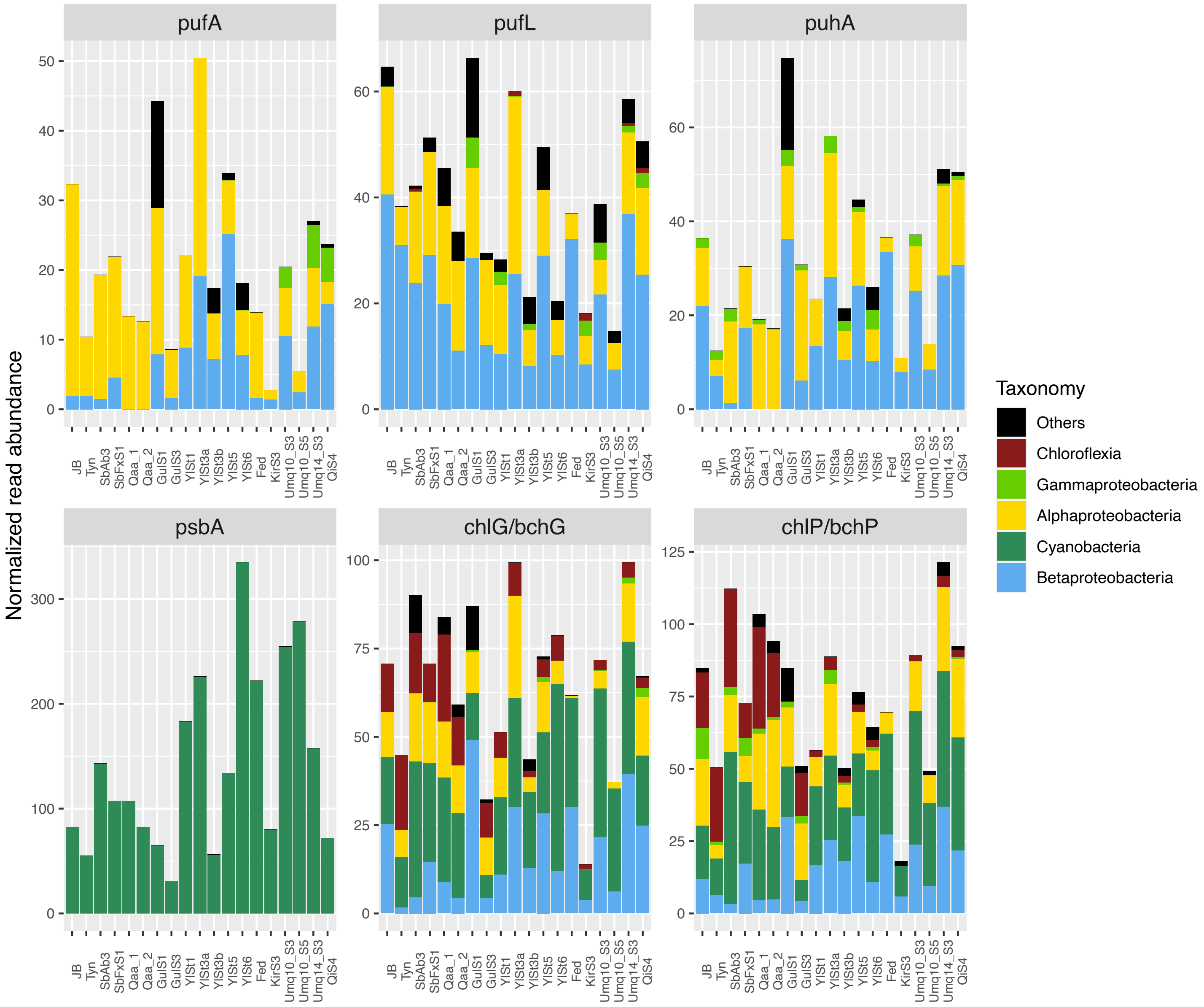

Figure S8. Read abundance of the genes responsible for photosystems.
Read abundance was normalized to the total read abundance of KEGG-annotated genes in each sample. *pufA*: light-harvesting complex 1 alpha chain; *pufL*: photosynthetic reaction center L subunit; *puhA*: photosynthetic reaction center H subunit; *psbA*: photosystem II P680 reaction center D1 protein; *chlG/bchG*: chlorophyll/bacteriochlorophyll *a* synthase; *chlP/bchP*: geranylgeranyl diphosphate/geranylgeranyl-bacteriochlorophyllide *a* reductase.

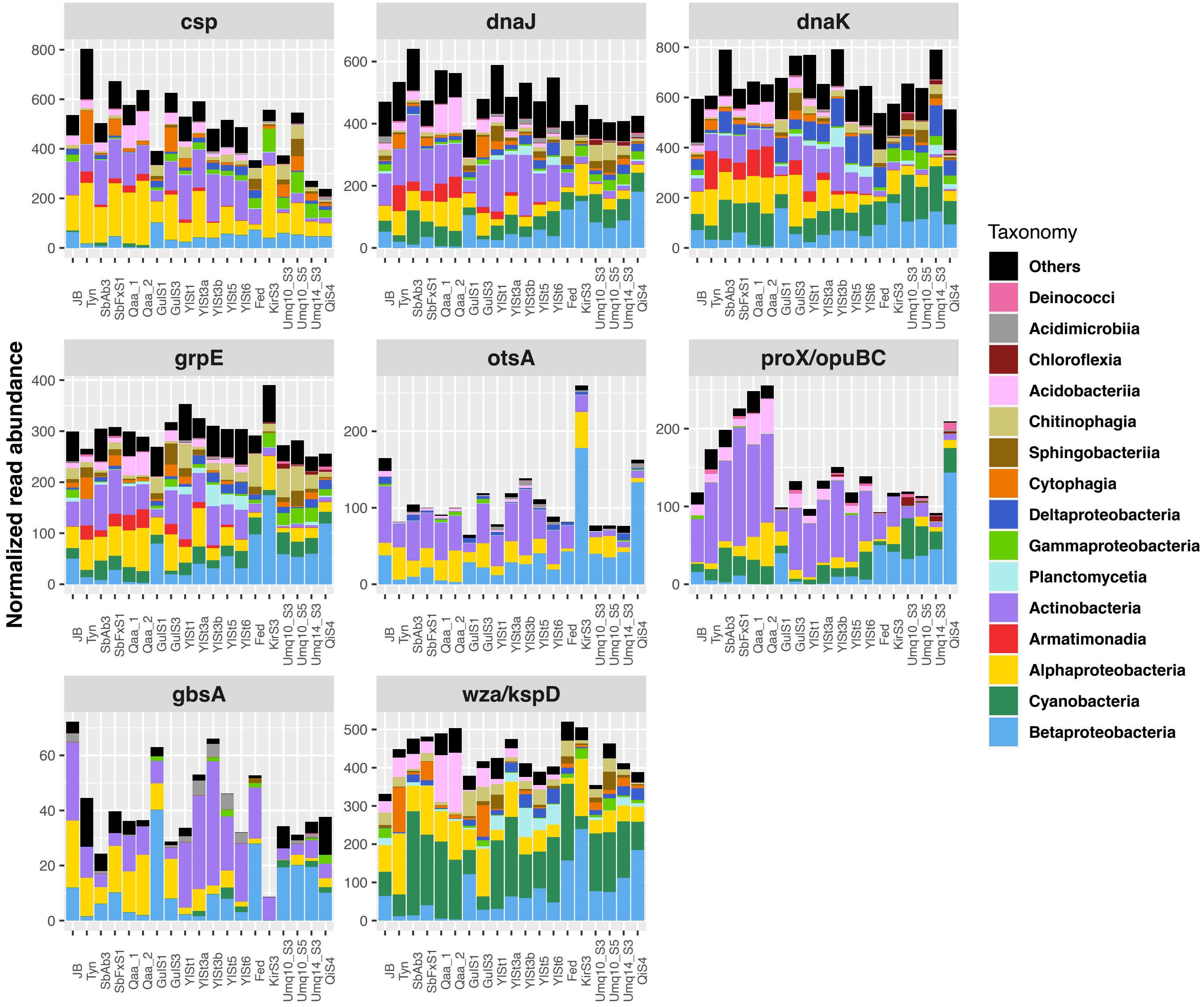

Figure S9. Read abundance of the genes involved in the psychrotolerance.
Read abundance was normalized to the total read abundance of KEGG-annotated genes in each sample. *csp*: cold-shock protein family; *dnaJ*: molecular chaperone DnaJ; *dnaK*: molecular chaperone DnaK; *grpE*: molecular chaperone GrpE; *otsA*: trehalose 6-phosphate synthase; *proX/opuBC*: glycine betaine and proline transport system substrate-binding protein; *gbsA*: betaine-aldehyde dehydrogenase; *wza/kspD*: polysaccharide export protein.

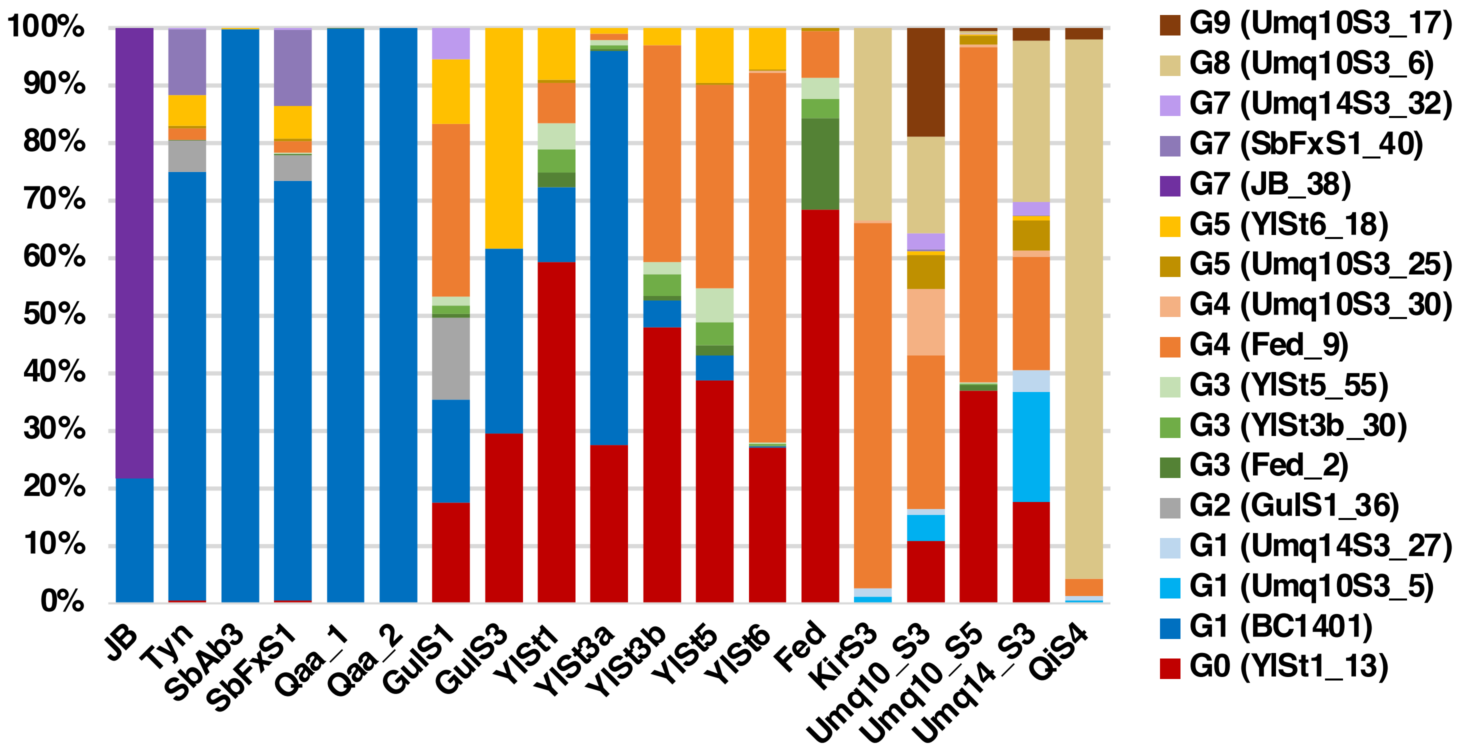

Figure S10. Composition of cyanobacterial lineages based on the read abundance of the metagenome-assembled genomes (MAGs).

Sixteen MAGs achieving highest quality scores (Completeness - 2x Contamination) and a draft genome of *Phormidesmis priestleyi* BC1401 were chosen as the references of 17 species-level (ANI ≥95%) cyanobacterial lineages. Composition was calculated based on the median value of read abundance mapped on the protein-coding regions (CDS) within each MAG/draft genome. CDSs which have homologues with ≥98% nucleotide identity were excluded from the calculation.
